# Defining Adequate Clinical Information in Chest X‐Ray Requests: A Delphi Study

**DOI:** 10.1002/jmrs.70145

**Published:** 2026-11-11

**Authors:** Athanasios Georgiopoulos, Madison Campbell, Tom Steffens, Chelsea Castillo

**Affiliations:** ^1^ Department of Diagnostic Radiology Princess Alexandra Hospital Brisbane Queensland Australia; ^2^ Centre of Online Health The University of Queensland Brisbane Queensland Australia

**Keywords:** Delphi technique, diagnostic imaging, education, radiography, reference standards, thoracic

## Abstract

**Introduction:**

Chest x‐rays (CXRs) are the most common radiographic examinations performed. Given their prevalence and the potential for associated stochastic effects, it is vital these examinations are justified through high‐quality requests. In the absence of published Australian guidelines defining adequate clinical information in CXR requests, this study aimed to develop a criteria standard reflecting radiologists' specific clinical information requirements to support optimal reporting.

**Methods:**

Following ethical approval (HREC/2025/QMS/122483), a three‐round electronic‐Delphi study of six consultant radiologists was conducted using Microsoft Forms. Round 1 invited open‐ended responses to create a list of what panellists required in CXR requests. Items from this list were rationalised into common categories for subsequent rounds. For Round 2, a dichotomous scale helped narrow the list of items using a 50% agreement threshold. Round 3 had panellists rank the remaining items from most useful to least useful. The final criteria standard consisted of the five most essential items.

**Results:**

The panel provided 54 items in Round 1. These were organised into 17 categories to avoid redundancy and support consensus building. In Round 2, six items passed the 50% agreement threshold. Consensus was gained in Round 3, and the five highest ranked items formed the criteria standard for information required in CXR requests.

**Conclusion:**

This study established a consultant radiologist‐derived standard for the clinical information required in CXR requests. Adoption of these criteria may improve request quality and support more informed radiology reporting, with future research evaluating the impact of targeted education on request and report quality.

## Graphical abstract

This e‐Delphi study developed a criteria standard identifying the clinical information that radiologists found essential in chest x‐ray requests to support optimal reporting. This criteria standard will be used for education of referrers to promote effective communication between them and medical imaging professionals. An AI tool (Microsoft Copilot) was used to support the development of the graphical abstract. The authors provided the design specifications, reviewed all outputs, and were solely responsible for the accuracy of the scientific content and final version of the figure.

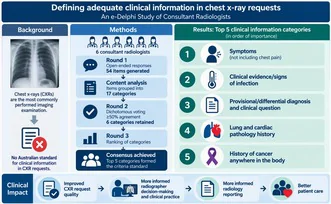

## 1 Introduction/Background

Chest x‐rays (CXRs) are the most common diagnostic examination undertaken in medical imaging departments [1]. They provide a plethora of information for a variety of symptoms and are amongst the first examinations undertaken to evaluate the heart and lungs specifically and overall patient health status generally [1]. Given the risk of stochastic effects associated with ionising radiation, justification of CXR examinations is a vital medico‐legal pillar of radiation safety for radiographers and healthcare organisations, as outlined in the Australian Radiation Protection and Nuclear Safety Agency's (ARPANSA) Radiation protection principles [2]. This justification occurs at multiple stages, initially by the referrer and subsequently by radiographers upon receiving the request. When requests lack necessary clinical information, justification by radiographers before performing the examination becomes more difficult. This can cause delays in completing patient imaging, negatively impacting workplace efficiency. Such inefficiencies can result in increased patient length of stay and unnecessary radiation [3].

Imaging requests are the primary form of communication connecting referrers with radiographers and radiologists [4, 5]. Without sufficient, relevant clinical information in CXR requests, it is challenging for radiographers to make decisions about the projections required to answer referrers' clinical questions. Additionally, lack of clinical information limits the reporting accuracy, confidence and clinical relevance of the radiology report which can lead to missed diagnoses [4, 5, 6, 7]. Ultimately, these issues can lead to negative impacts on patients' healthcare journeys. Ensuring adequate clinical information is included in CXR requests enables the most efficient pathway from request submission to radiologist image interpretation and reporting [4, 5, 6, 7, 8].

Current Australian and international guidance highlights the importance of providing sufficient clinical details in imaging requests to support appropriate examination selection and interpretation. In the United Kingdom, the Royal College of Radiologists (RCR) created the iRefer guidelines to support appropriate imaging requests determined by supporting evidence, consideration of radiation dose, and cost‐effectiveness [9]. In Australia, the Royal Australian and New Zealand College of Radiologists (RANZCR) has adopted the RCR's iRefer guidelines [9, 10]. These guidelines recommend that the reason for the request must be clearly stated with sufficient clinical details. Similarly, Queensland Health guidance specifies the inclusion of relevant clinical notes, medical conditions and the reason for the procedure [11]. These international and local recommendations are similar in the fact that they describe the requirement for sufficient clinical information at a broad level without defining a specific set of information that should be consistently documented for requests. The lack of specificity in current recommendations highlights the importance of gaining expert consensus regarding the specific clinical information required in CXR requests.

There are no current guidelines which stipulate what constitutes ‘adequate’ clinical information in CXR requests. Given that the aforementioned local and international recommendations mention ‘adequate’ clinical information as a requirement for imaging requests, this can lead to confusion for referrers and medical imaging professionals. In a four‐stage e‐Delphi study, Castillo et al. [8] were successful in creating a specific criteria standard for adequate clinical information in commonly requested acute computed tomography (CT) examinations, specific to Australian practice. This study seeks to emulate this approach to create a similar standard specific to CXR requests. This study aimed to create a criteria standard reflecting radiologists' specific clinical information requirements in CXR requests to support optimal reporting.

## 2 Methods

Ethics approval was obtained from Metro South Research Ethics prior to commencing the study (HREC/2025/QMS/122483). A three‐round e‐Delphi study was conducted to gain consensus from consultant radiologists regarding the specific clinical information considered essential for inclusion in CXR requests to support optimal reporting. The e‐Delphi method of obtaining information to form a consensus is useful in synthesising information for this issue, where there is limited research [12, 13]. This study type has a proven track record for gathering insights from a panel of subject matter experts when creating guidelines in healthcare [12, 13].

The panel of consultant radiologists was recruited from the Princess Alexandra Hospital (PAH) which is a tertiary and quaternary teaching hospital in Brisbane, Queensland, Australia. Part of the Metro South Health Service, the PAH manages patients with a diverse range of clinical presentations and levels of complexity. The PAH provides a plethora of diagnostic medical imaging services, including CXRs, for inpatient, outpatient and emergency clinical settings. Given the broad range of clinical presentations for which CXRs are requested, this study setting was deemed appropriate.

### 2.1 Recruitment

A voluntary sampling approach was used to recruit potential participants to the expert panel. All 38 consultant radiologists at the PAH were emailed a participant information sheet (PIS) outlining the study's purpose and methods, inviting them to participate in the survey. This email included the Round 1 survey link and was sent in November 2025. From the 38 consultant radiologists at the PAH, six consented to partake in the e‐Delphi study (approximately 16%). Although the number of participating consultant radiologists was relatively small, this was considered appropriate given the specialised nature of the target population and the deliberate recruitment of individuals with relevant clinical expertise. This Delphi methodology prioritises the selection of knowledgeable and relevant experts rather than statistical representativeness. The six panellists consisted of consultant radiologists ranging in experience from 6 to 22 years. Despite maintaining anonymity between the consultant radiologist panellists, it was not possible for the panellists to be anonymous to the researchers due to the Delphi method used. Despite this, the number of participants falls within the appropriate range typically required to form an expert panel and achieve consensus, as described in previous Delphi studies [14]. The retention rate was 100% over the three survey rounds (attrition rate of 0%).

### 2.2 e‐Delphi Survey Rounds

This study consisted of three electronic online survey rounds using Microsoft Forms. Each round was open to responses for 1 month, with participants given two reminders during each round. The language used in the survey items was informed by a similar previously reported e‐Delphi study [8]. As the survey items were simple in nature, a review of these items was not considered necessary. Round 1 consisted of an open‐ended question inviting the expert panel to answer using free‐text responses. This allowed the collection of qualitative data and allowed the expert panel to provide detailed responses without restriction [15]. The first round was conducted in November 2025. The six panellists who consented to participate in the study were asked to provide a list of clinical information and indications they find useful for enabling optimal reporting of CXR examinations. The responses were analysed by four radiographers, all part of the research team. An inductive content analysis approach was used, as described in Hsieh and Shannon [16]. Initial analysis involved synthesis and rationalisation of similar responses into categories and the removal of duplicate responses. An example of the analysis is outlined below:

1. Duplicate items were identified and removed.
2. Similar terms were grouped together. For example, ‘history of smoking’ and ‘smoking or vaping history’, were combined into the category ‘smoking/vaping history’.
3. Responses with similar meanings but different terms were also rationalised and categorised together. For example, ‘desaturation’ and ‘temperature/fever’ were combined into the category ‘clinical evidence/signs of infection’.

This process was conducted by all four researchers independently, with discrepancies between individual assessments resolved through discussion and consensus.

In the second round, the six panellists were provided with a summary of all first‐round responses, including how these responses were collated and organised. This transparency allowed panellists to raise any concerns about the organisation of Round 1 responses. No concerns were raised. The Round 2 survey link was included in this email. Round 2 asked the six panellists whether they considered the categories identified in Round 1 to be essential for inclusion in requests to enable optimal reporting of CXRs. For each category, panellists were provided with a dichotomous choice (Yes/No). Round 2 was emailed to panellists in January 2026. All categories deemed essential by 50% or more of the panellists were included in Round 3.

A 50% or more agreement was determined as the minimum threshold for progression to the third round as it has been used in previous Delphi studies involving the development of referral criteria. For example, Chang et al. [17] used this threshold to determine which referral criteria progressed from round one to two. Similarly, in a Delphi study developing criteria for an exercise intervention, Behan et al. [18] specified that items receiving less than 50% agreement would be excluded from progression to the next round. The authors used this threshold to remove items with insufficient expert support while allowing potentially relevant criteria to undergo further consideration in subsequent rounds. This study used the same threshold of 50% agreement to indicate the criteria items that warrant further expert consideration in the third round.

The third round's email provided the six panellists with a summary of Round 2 and the link to the Round 3 survey. In this round, panellists were asked to rank the remaining items from Round 2 according to their perceptions regarding each item's impact on optimal CXR interpretation and reporting. The results from this round were used to establish a criteria standard of essential clinical details for inclusion in CXR requests for optimal reporting. Round 3 also gave panellists the ability to add free‐text comments regarding their rankings. This enabled panellists to suggest final refinements to the wording and raise any concerns, ensuring the accuracy of the final consensus. This round was emailed to panellists at the end of March 2026. Overall, the study lasted for 6 months from November 2025 to the end of April 2026.

Consensus in the third round was achieved when the interquartile range (IQR) was equal to or less than two, a measure used as an indicator of consensus in previous similar studies [13, 19]. Smaller IQR values indicate greater concentration of responses and therefore an indicator of greater agreement amongst the panellists [20]. A summary of the e‐Delphi process can be seen in Figure 1 below.

**FIGURE 1:**
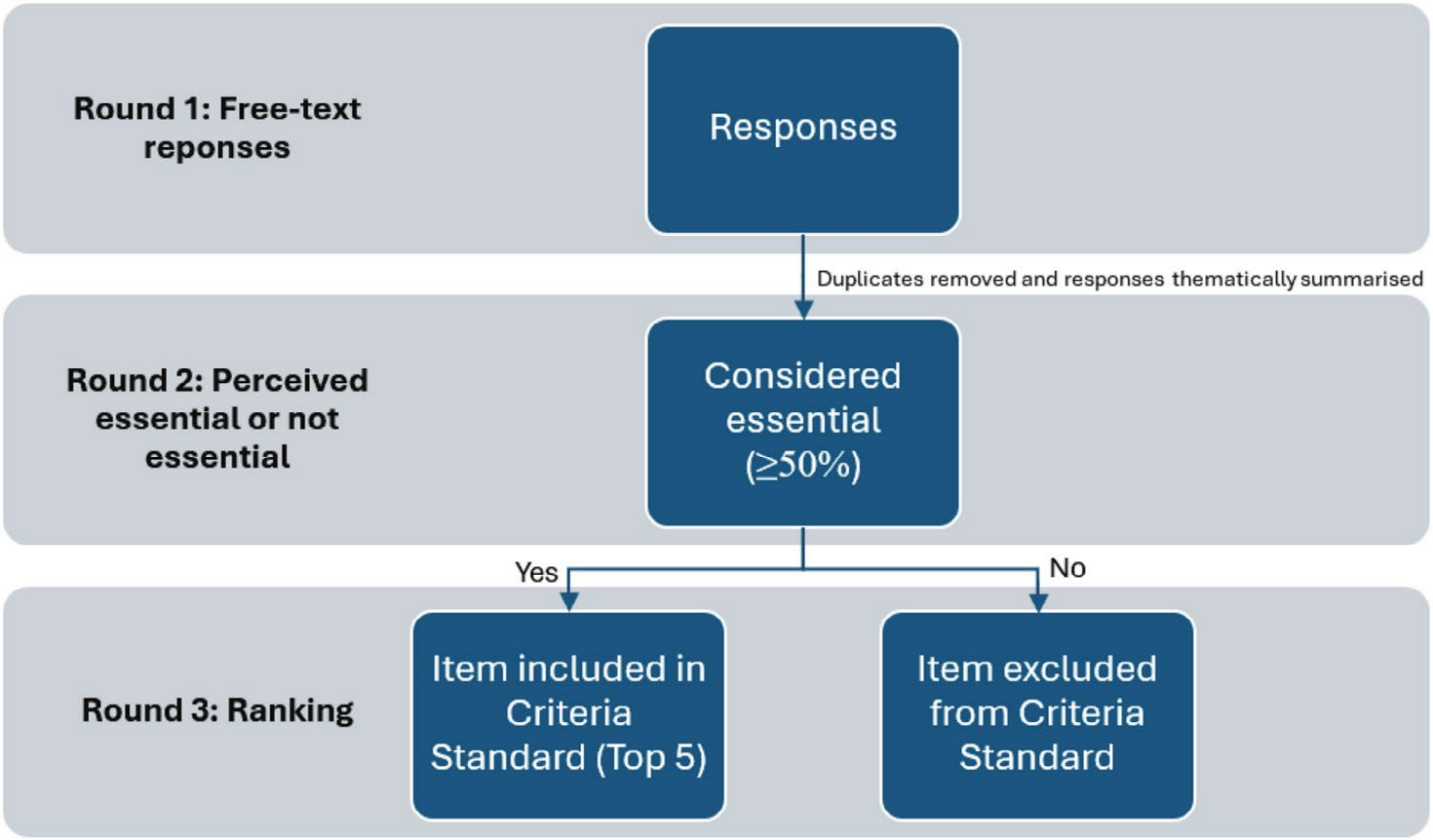
Summary of e‐Delphi process.

## 3 Results

Thirty‐eight consultant radiologists were invited to participate in the study, of whom six participated in the first round of the survey to form the expert panel. This number of participants falls within the appropriate range typically required to form an expert panel and achieve consensus, as described in previous Delphi studies [14]. The retention rate was 100% over the three survey rounds.

The 54 responses provided by the panellists in Round 1 were analysed and rationalised into 17 categories of clinical information and included in Round 2. Rationalisation included the collating of similar/identical items into categories. For example, the responses ‘any cancer history’ and ‘history of prior malignancy’ were combined into the one category ‘history of cancer anywhere in the body’. Additionally, terms with similar meanings were grouped even if different terms were used in the panellists' responses. For example, responses like ‘clinical question’ and ‘clinical concern’ were grouped into the category ‘provisional/differential diagnosis and clinical question’. This analysis and categorisation can be seen in Table 1. Out of the 17 categories created from Round 1, six met the 50% threshold to be included in Round 3 as seen in Table 2.

**TABLE 1:** Round 1 results with examples of categorisation of responses.

Category	Example from text
Provisional/differential diagnosis and clinical question	‘Specific questions for which answers are being sought’ ‘Is the study for ruling in a diagnosis, ruling out a diagnosis or because they don't know what is wrong with the patient (diagnostic dilemma)’ ‘Clinical concerns regarding pulmonary oedema, myocardial infarction, spine, and/or free gas’
Symptoms (not including chest pain)	‘Any infective symptoms’ ‘Presenting complaint’ ‘Clinical symptoms for current presentation’
Characteristics of symptoms (not including chest pain)	‘Duration of presenting symptom’
Any chest pain	‘Any chest pain’
Characteristics of chest pain	‘Pain – where, when, type of pain’ ‘Where chest pain is located, nature of pain, pleuritic etc.’ ‘Site and characteristics of chest pain’
Clinical evidence/signs of infection	‘Clinical findings’ ‘How sick is the patient (recent bloods, temp, etc.)’ ‘Desaturation, sputum’
Lung and cardiac pathology history	‘Any cardiac history’ ‘Any asthma/COPD history’ ‘Relevant chest PMHx’
Smoking/vaping history	‘Smoking/vaping history’
Surgical history in the thorax only	‘Any relevant surgeries (old/recent)’ ‘Relevant history (e.g., PSHx)’
Surgical history in the thorax and abdomen only	
Surgical history in any part of the body	
History of cancer in thorax only	‘History of primary malignancy and metastasis’ ‘Relevant extra‐thoracic PMHx (e.g., cancer, treatments)’ ‘H/O prior malignancy and treatment – particularly if thoracic including chest wall, breast, etc.’
History of cancer in thorax and abdomen only	
History of cancer anywhere in the body	
Any external imaging (chest x‐ray only)	‘Any external imaging’ ‘Details of prior imaging if not done at PA before – like name search etc.’ ‘Known echo abnormality’
Any external imaging of the thorax only (includes CT and other modalities)	
Any external imaging of anywhere in the body (all modalities)	

**TABLE 2:** Round 2 results.

Category of clinical information	Panellists' response		Percentage of panellists that deemed the category as essential
	Yes	No	
Provisional/differential diagnosis and clinical question	6	0	100%
Symptoms (not including chest pain)	5	1	83%
Characteristics of symptoms (not including chest pain)	2	4	33%
Any chest pain	2	4	33%
Characteristics of chest pain	2	4	33%
Clinical evidence/signs of infection	3	3	50%
Lung and cardiac pathology history	4	2	67%
Smoking/vaping history	3	3	50%
Surgical history in the thorax only	2	4	33%
Surgical history in the thorax and abdomen only	1	5	17%
Surgical history in any part of the body	1	5	17%
History of cancer in thorax only	1	5	17%
History of cancer in thorax and abdomen only	2	4	33%
History of cancer anywhere in the body	4	2	67%
Any external imaging (chest x‐ray only)	1	5	17%
Any external imaging of the thorax only (includes CT and other modalities)	2	4	33%
Any external imaging of anywhere in the body (all modalities)	0	6	0%

*Note:* Percentage of panellists who identified each category as essential. Green rows: Categories that progressed to Round 3 of the survey (met the 50% acceptance threshold). Red rows: Categories that did not progress to Round 3 of the survey (did not meet the 50% acceptance threshold).

In Round 3, panellists were asked to rank the six categories which reached the 50% threshold in Round 2 from most to least impact on their reporting of CXR examinations. The outcome of Round 3 is shown in Table 3. The highest‐ranking categories (in order) were symptoms (not including chest pain), clinical evidence/signs of infection (e.g., recent bloods, temperature, desaturation, sputum), provisional/differential diagnosis and clinical question, lung and cardiac pathology history, history of cancer anywhere in the body, and smoking/vaping history (which was not included in the final criteria standard). A consensus was reached amongst the panellists which was demonstrated as each category had an IQR of two or less, indicating high consistency around the central value. No further items were suggested and no concerns raised by the panellists in the free‐text section of Round 3.

**TABLE 3:** Round 3 results.

Category	Ranking						
	1st choice	2nd choice	3rd choice	4th choice	5th choice	6th choice	Median ranking (IQR)
Symptoms (not including chest pain)	4	—	1	1	—	—	1 (2)
Clinical evidence/signs of infection (e.g., recent bloods, temperature, desaturation, sputum)	1	3	—	1	1	—	2 (2)
Provisional/differential diagnosis and clinical question	1	1	2	1	—	1	3 (2)
Lung and cardiac pathology history	—	2	1	2	1	—	3.5 (2)
History of cancer anywhere in the body	—	—	2	—	3	1	5 (2)
Smoking/vaping history	—	—	—	1	1	4	6 (1)

*Note:* Panellists' ranking of the remaining categories from most impactful to least impactful on optimal CXR interpretation and reporting. Green rows: Categories that were included in the criteria standard. Red rows: Categories that were not included in the criteria standard.

Abbreviation: IQR, interquartile range.

Research into working memory suggests that approximately three to five meaningful elements can be actively maintained at one time, particularly when processing novel or complex information [21]. Therefore, the criteria standard was limited to five criteria to minimise the cognitive load for referrers while ensuring that the necessary information required for appropriate CXR examination justification and radiologist interpretation was captured. Ultimately, it was anticipated that distilling the list to five items would facilitate the creation of a concise, practical criteria standard.

## 4 Discussion

Our e‐Delphi study achieved its aim to develop a criteria standard for CXR requests reflecting radiologists' clinical information requirements to support optimal reporting and positively impact radiographer practice. The criteria standard as decided by consensus of the expert panel consisted of the following: symptoms (not including chest pain); clinical evidence/signs of infection (e.g., recent bloods, temperature, desaturation, sputum); provisional/differential diagnosis and clinical question; lung and cardiac pathology history; and history of cancer anywhere in the body. The success of this study enables these criteria to be shared confidently as evidence‐based advice for referring clinicians, as they are drawn from expert consensus, not arbitrary preferences of individual radiologists.

In Round 1, panellists listed 54 individual items they would find useful in CXR requests to facilitate optimal reporting, a number which even after rationalisation still resulted in 17 distinct elements. This finding reinforces the notion that radiologists have a need for more information in CXR requests than what has previously been provided. This finding mirrors a study which used a similar method to create a criteria standard for CT requests in the emergency department [8].

The authors anticipate that the creation of a relatively concise, specific criteria standard as a result of this study will encourage referrers to implement it as part of their requesting practice. Existing guidelines such as those published by The Royal Australian and New Zealand College of Radiologists (RANZCR) recommend ‘sufficient clinical detail’ to justify the performance of examinations [22]. Broad terms such as ‘sufficient’ give referrers little confidence as to whether their requests meet requirements. This was reflected in a study conducted by Borgen et al. [23] which found that only 19.5% of surveyed clinicians stated they used available guidelines. This low level of engagement with current guidelines may be due to referrers' confusion around the meaning of current, subjective requesting guidelines. It is hoped that the clarity provided by the standard in this study will enable greater compliance.

It is likely that the criteria standard created as an outcome of this study will also assist radiographers in selecting the appropriate radiographic projections [5]. In particular, the inclusion of items around cardiac and oncology history is useful, as both of these necessitate the inclusion of a lateral projection. Additionally, when cardiac history is mentioned, a postero‐anterior projection is strongly preferred as it enables accurate measurement of the cardio‐thoracic ratio, a key suggestive indicator for heart failure. The inclusion of information around existing lung pathology will also facilitate radiographer decisions around appropriate exposure technique, as conditions such as pneumonia and emphysema are known to impact lung density and require technical adjustment by radiographers.

As CXR examinations are widely requested [1], we anticipate the creation of this criteria standard may have a considerable impact on the quality of clinical information provided in future requests, as well as improve referrers' adoption and confidence in using criteria standards to guide requesting. This is supported by a recent study which assessed the effectiveness of an education program based on request criteria standards on improving acute CT request quality [24]. The study concluded that overall, by clearly defining the request requirements specific to examinations, referrers are more likely to comply with criteria standards.

The criteria standard created in this study will also assist radiographers in determining whether future CXR requests are justified. By defining the items radiologists consider essential, this study has by extension created a threshold standard that radiographers can apply. All requests which fulfil the standard can safely be deemed appropriate and justified by radiographers. On occasions when radiographers receive requests which do not contain all the specific, essential information, the criteria standard will provide a basis for radiographer conversations with referrers around justification of the imaging, rather than merely relying on their personal judgement [25].

Currently, the Medicare Benefits Schedule regulatory framework does not prescribe what clinical information should be included in medical imaging requests [26]. This study determined that the clinical question was one of the major categories that the panellists found necessary on CXR requests. This finding is supported by the RANZCR's recommendation that the clinical question should be included to assist imaging providers, such as radiographers, in determining the appropriateness of the requested examination and ensuring that the clinical question is addressed [22]. This recommendation further supports the inclusion of a clearly defined clinical question within the CXR request criteria standard created in this study and highlights the importance of achieving this study's aim to move away from the broad requirements currently in place and create specific criteria standards.

## 5 Implications for Practice

The criteria standard developed in this e‐Delphi study can be used to support effective communication between referrers and reporting radiologists regarding CXRs. It is anticipated it will be utilised as an educational tool to guide referrers in ensuring their CXR requests are justified, thereby reducing unnecessary imaging and delays due to queries from radiographers. In a survey conducted by Thormahlen et al. [27], it was determined that over 85% of referrers used the radiology reports, and that clinical history was a crucial element in constructing a good radiology report for radiologists. This study also mentioned that a majority of referrers believe that the quality of reports is linked to the amount of clinical history in requests [27]. This suggests that referrers may be open to an education program around this topic. As education around radiology requesting is known to be lacking in Australian university undergraduate medical degrees [28, 29], it is likely junior doctors will be motivated and receptive. Therefore, it is likely the dissemination of this criteria standard could improve the confidence and skills of referrers when requesting CXRs through education.

Similarly, radiographers could use this criteria standard to add further clinical information to requests at the time of examination; this may be achieved through further discussions with patients or referrers or interrogation of patients' electronic medical record. Using the criteria standard in this way will assist radiographers to comply with their duty of care to confirm and tailor each examination, as prescribed in Domain 1.7 of the Medical Radiation Practice Board of Australia's Professional Practice Capabilities [30]. Taking a clinical history not only is a duty of care of radiographers, but evidence suggests that it enhances radiographers' job satisfaction [31].

Ultimately, implementation of these criteria into CXR request guidelines, referrer education programs, electronic requesting systems and radiographer‐patient interactions could enhance communication between referrers and medical imaging professionals and encourage more consistent imaging practices. This improved communication has been shown to promote patient‐centred care by improving the accuracy, diagnostic confidence and clinical relevance of reports [7, 32].

## 6 Limitations and Future Research

This e‐Delphi study had several limitations. Panellists were selected from a single, albeit large, tertiary and quaternary referral hospital in Queensland. The lack of diverse panel characteristics limits the generalisability of the results to other clinical settings such as private clinics, where less acute presentations are more prevalent. Despite six expert panellists being enough to gain consensus on the criteria standard, the sample size is small and therefore may not reflect the perspectives of consultant radiologists nationally. To address these limitations, future research could be conducted to improve the generalisability and national applicability of the criteria standard. This could be achieved by broadening the e‐Delphi study to consultant radiologists across multiple sites, including different states and territories of Australia. This could serve to validate the proposed criteria standard or serve to strengthen it by adding to the evidence base.

The next phase in this research will comprise an evaluation of the effectiveness of the criteria standard, by measuring what effect education of referrers has on the appropriateness of CXR requests and adherence to the criteria standard. A further study will look at whether implementing this criteria standard leads to improved report quality.

## 7 Conclusion

This e‐Delphi study aimed to develop a criteria standard for the clinical information required in CXR requests, based on the requirements of consultant radiologists. Through the three rounds of this study, the aim was achieved. Future research will investigate the effect of targeted education regarding the established CXR request criteria standard, with associated evaluation of its impact on request quality and radiology report quality.

## Funding

The authors have nothing to report.

## Conflicts of Interest

The authors declare no conflicts of interest.

## Data Availability

The data that support the findings of this study are available from the corresponding author upon reasonable request.
